# Evaluation of GATA3 and GCDFP15 Expression in Triple Negative Breast Cancers

**DOI:** 10.30699/IJP.2023.561917.2969

**Published:** 2023-03-23

**Authors:** Mahsa Ahadi, Afshin Moradi, Elham Rabiee, Fatemeh Pourmotahari

**Affiliations:** 1 *Men’s Health and Reproductive Health Research Centre, Shahid Beheshti University of Medical Sciences, Tehran, Iran*; 2 *Department of Pathology, School of Medicine, Shahid Beheshti University of Medical Sciences, Tehran, Iran*; 3 *Clinical Research and Development Center, Shahid Modarres Hospital, Shahid Beheshti University of Medical Sciences, Tehran, Iran*

**Keywords:** Breast cancer, GATA-3, GCDFP-15, Triple-negative breast cancer

## Abstract

**Background & Objective:**

Breast cancer is one of the most common cancers in the world. There are some different types of breast cancer and triple-negative breast cancer is the type in which no receptors for estrogen, progesterone, and human epidermal growth factor receptor-2 are expressed. Identifying factors that can facilitate the diagnosis of triple-negative breast cancer is important. In this study, we decided to investigate the expression of GATA3 and GCDFP15 genes in triple-negative breast cancers.

**Methods:**

This is a retrospective descriptive-analytical study that was performed on 50 specimens of samples of triple-negative breast cancer. Data including age and sex, tumor grade, tumor size, types of invasion, GATA-3, and GCDFP-15 were assessed.

**Results:**

The mean age of the patients was 48.3±14.17 years. Of the total specimens, 46% were positive for GCDFP15 and 90% were positive for GATA-3. The intensity of GATA3 was evaluated and it was observed that 33(73.3%) of the cells were strongly stained and 12(26.7%) were weakly stained. There were no relationships between GATA-3 and GCDFP-15 with tumor characteristics.

**Conclusion:**

GATA-3 and GCDFP-15 may serve as diagnostic markers for triple-negative breast cancers and GATA-3 seems to be more reliable.

## Introduction

With much more than 1 in 10 newly diagnosed cancers annually, breast cancer is the most common cancer in women. It is the second most frequent cancer-related death among women worldwide (1-3). Using immunohistochemistry (IHC), the three hormone receptors for estrogen receptor (ER), progesterone receptor (PR), and human epidermal growth factor receptor-2 (HER2) are first identified in breast cancer tumors to determine the type of breast cancer (4-6). Triple-negative breast cancers are among six types of breast cancer that have no receptors for ER, PR, and HER2 (7). There is a relationship between age and triple-negative breast cancers (8, 9).

One of the six zinc finger transcription proteins in the GATA family that identify a particular nucleotide sequence in the promoter region of target genes is GATA binding protein 3 (10). The multi-specific and effective immunohistochemical breast differentiation marker GATA-3 has been discovered (11). It was revealed that GATA-3 has a high positive predictive value (96.2 percent) for identifying the origin of malignant effusions due to breast cancer (12). Evidence indicates that GATA-3 is more sensitive than the previously mentioned markers mammaglobin and gross cystic disease fluid protein 15 (GCDFP-15) and is expressed in triple-negative breast cancer (13). Previous studies indicate the expression of the GCDFP-15 gene on breast apocrine glands in different malignancies. Due to the specific expression of this gene in breast tissue differentiation in women, it is used as a specific marker in immunohistochemistry to evaluate and differentiate primary breast cancer from metastatic carcinoma and to find the origin of the tumor in metastatic breast cancer. The expression of GCDFP-15 is regulated by the androgen receptor (AR) and in general, due to limited studies, there is no detailed information about the function of this gene in breast tissue (14).

Limited studies have investigated the diagnostic value of this gene in triple-negative breast cancer and compared its diagnostic value with GATA-3, and this issue requires more detailed and extensive studies. Regarding the limitation of previous studies in the field of introducing new diagnostic markers for the diagnosis of triple-negative breast cancer, in this study, the diagnostic value of GATA-3 and GCDFP-15 markers was investigated.

## Material and Methods

This retrospective descriptive-analytical study was performed on 50 samples of triple-negative breast carcinoma which were collected in paraffin from the pathology laboratory of Shahada-e-Tajrish Hospital. The data collection was performed by a single random method.

Diagnosis of carcinoma type and tumor grading was done by two expert pathologists. Patients’ data including age and sex, tumor grade, tumor size, lymphovascular invasion, lymph node involvement, GATA-3, and GCDFP-15 related markers (including the presence of each marker and their intensity after staining) were evaluated. Five normal samples were selected as the control group.

**Immunohistochemical Study**

First, 4-micron sections were prepared from the paraffin blocks with a sterile microtome blade and placed on slides covered with poly-L-lysine glue. After deparaffinization, the sample was subjected to antigen recovery by heating in 10 mM citric acid solution (pH = 6) for 30 minutes. In order to inhibit the activity of internal peroxidases, the tissue sections were incubated with 0.03% hydrogen peroxide for 10 minutes and then the tissues were incubated with serum blocker solution for 10 minutes at room temperature. Primary antibodies were against GATA3 and GCDPF-15. The tissue sections were first placed with the primary antibody for one night at refrigerator temperature, and the next day, they were incubated with the secondary antibody for one hour at room temperature. Then the tissue sections were washed with PBS and after passing through Xylitol and alcohols in different dilutions (opposite to the first step), they were mounted with a lamel. An optical microscope was used for evaluation. To quantify the data obtained from Immunohistochemical staining, the intensity staining was graded as weak or strong (15). For GATA3 Only nuclear staining was regarded as positive. In fact, the distribution was recorded as negative (<5% of tumor cells stained), +1(5%-25%), +2(26%_50%), +3(51%_75%) or +4(>75%) (16). Also, the intensity of staining was graded as weak and strong. For the GCDF-15 marker, the percentage of cells, whose cytoplasm was stained, was divided into three groups: focal (less than 10% of cells were stained), patchy (10 to 50% were stained), and diffuse (more than 50% of cells were stained). In addition, these cells were graded according to the color intensity into weak, medium, and strong. Finally, if they were patchy or diffuse with medium or strong intensity, the result was considered positive, and if focal or weak, it was considered negative (17).

The nuclear grade was assessed based on the Nottingham Histological Grading Scale. The Nottingham Histological Grading Scale rates the degree of tube development, nuclear pleomorphism, and mitosis on a scale of 1 to 3 (18). 

**Ethical Issue**

This study was approved by the ethical committee of Shahid Beheshti Medical University (IR.SBMU.RETECH.REC.1399.854)

**Statistical Analysis**

Mean, standard deviation, frequency, and percentage were used to describe the data. The Chi-square test and Fisher's exact test were used to compare qualitative variables between the two groups. All analyzes were performed by SPSS 25.0 (SPSS Inc., Chicago, Ill., USA). P-values less than 0.05 were considered statistically significant**.**

## Results

The aim of this study was to investigate the expression level of GCDFP15 and GATA3 in triple-negative breast cancer. Fifty paraffin blocks were collected from triple-negative breast carcinoma samples. The mean age of the patients was 48.3±14.17 years with a range of 27 to 88 years. The GATA3 marker in terms of the rate of cells that stained, the intensity of the cells staining, and the GCDFP15 marker were evaluated. Of the total number of samples, 27 (54%) samples were negative for GCDFP15 and 23 (46%) were positive for GCDFP15. About GATA3, 5(10%) of the samples were stained less than 5% of cells, 3 samples (6%) were stained 5-25%, 5(10%) samples were stained 26-50% of cells, 14 (28%) samples were stained 27-75%, and 23 (46%)samples were stained more than 75% of cells. Table 1, shows the relationship between GATA3 intensity staining, GCDFP15, and tumor parameters. 

**Table 1:** The relationship between GATA3, GCDFP15, and tumor parameters

		GATA3		P-value
		Strong,N(%)	Weak,N(%)	
GCDFP15	Negative	15 (65.2%)	8 (34.8%)	0.208*
	Positive	18 (81.8%)	4 (18.2%)	
Nuclear Grade	2.00	9 (75%)	3 (25%)	>0.999**
	3.00	24 (72.7%)	9 (27.3%)	
Lymph node involvement	No	21 (67.7%)	10 (32.3%)	0.287**
	Yes	12 (85.7%)	2 (14.3%)	
Lympho vascular invasion	Negative	18 (69.2%)	8 (30.8%)	0.467*
	Positive	15 (78.9%)	4 (21.1%)	

*P-value based on Chi-square

**P-value based on Fisher Exact test

Table 2 shows our assessment the age and histologic grade in strong and weak stained samples. There was a statistically significant difference between samples over 50 years old and under 50 years old regarding staining (*P*=0.024).

Tables 3 and 4 show the tumor-related factors based on GATA3 stained percentage. There was no statistically significant difference between these variables with the percentage of stained cells.

**Table 2:** The relationship between GATA3 staining and age and histological grade

		GATA3.Intensity				
		**Strong**		**Weak**		P-value
Age	<50Yrs	15 (60.0%)		10 (40.0%)		0.024*
	>=50Yrs	18 (90.0%)		2 (10.0%)		
Histologic grade	1.00	1 (50.0%)		1 (50%)		0.192**
	2.00	11 91.7%)		1 (8.3%)		
	3.00	21 (67.7%)		10 (32.3%)		

*P-value based on Chi-square

**P-value based on Fisher’s Exact test

**Table 3:** The percentage of stained cells with some variables

		GATA3					
		<5%	5-25 %	26-50 %	51-75 %	> 75%	P-value
Nuclear grade	2.00	0 (0.0%)	0 (0.0%)	1 (7.7%)	5 (38.5%)	7 (53.8%)	0.071
	3.00	5 (13.5%)	3 (8.1%)	4 (10.8%)	9 (24.3%)	16 (43.2%)	
GCDFP15	Negative	4 (14.8%)	2 (7.4%)	3 (11.1%)	6 (22.2%)	12 (44.4%)	0.372
	Positive	1 (4.3%)	1 (4.3%)	2 (8.7%)	8 (34.8%)	11 (47.8%)	
Age	<50Yrs	3 (10.7%)	3 (10.7%)	3 (10.7%)	9 (32.1%)	10 (35.7%)	0.348
	>=50Yrs	2 (9.1%)	0 (0.0%)	2 (9.1%)	5 (22.7%)	13 (59.1%)	
Size	<=5	5 (15.2%)	2 (6.1%)	4 (12.1%)	6 (18.2%)	16 (48.5%)	0.419
	>5	0 (0.0%)	1 (10.0%)	0 (0.0%)	5 (50.0%)	4 (40.0%)	
Lymph node involvement	No	4 (11.4%)	3 (8.6%)	5 (14.3%)	10 (28.6%)	13 (37.1%)	0.557
	Yes	1 (6.7%)	0 (0.0%)	0 (0.0%)	4 (26.7%)	10 (66.7%)	
Lympho vascular invasion	Negative	4 (17.4%)	2 (8.7%)	2 (8.7%)	6 (26.1%)	9 (39.1%)	0.759
	Positive	1 (5.0%)	1 (5.0%)	2 (10.0%)	5 (25.0%)	11 (55.0%)	

**Table 4:** The relationship between the GCDFP15 positivity and the tumor related factors

P-value*	GCDFP-15 Positive	GCDFP-15 Negative	Total		
	23	27	50		**Total**
**0.027**	9(39.1)	19(70.4)	28	<50 years	**Age**
	14(60.9)	8(29.6)	22	>=50 years	
**0.467**	13(73.7)	21(84.0)	34	<=5	**Size of lesion**
	5(26.3)	4(16.0)	9	>5	
**0.313**	8(44.4%)	15(60%)	23	Negative	**Lymph Vascular Invasion**
	10(55%)	10(40%)	20	Positive	
**0.431**	1(8.7)	0(0.0)	1	I	**Histological grade**
	6(26.1)	6(22.2)	12	II	
	16(65.2)	21(77.8)	37	III	
**0.325**	7(30.4)	5(18.5)	12	II	**Nuclear grade**
	16(69.6)	22(81.5)	38	III	
**0.078**	9(50)	19(76)	28	Negative	**Lymph node involvement**
	9(50)	6(24)	15	Positive	

*Based on Fisher's exact test

## Discussion

In this study, we evaluated the expression of GATA3 and GCDFP15 in triple-negative breast cancers. We assessed 50 specimens of patients who were involved with triple-negative breast cancers. It was observed that the mean age was 48.30±14.17 years. Out of these specimens, 23 (46.0%) were GCDFP15 positive. GATA-3 staining showed that 10% of specimens were negative (stained less than 5% of cells), 6% was stained 5-25% (+1), 10% was stained 26-50% (+2), 28% was stained 51-75% (+3), and 46% was stained more than 75% (+4).33 (73.3%) patients had strong GATA-3 intensity and 12 (26.7%) had weak GATA-3 intensity.

There was no association between GATA3 expression and lymphovascular invasion, lymph node involvement, nuclear grade, and GCDFP15 presentation. In terms of GATA-3 intensity, it was found that there was a significant association between age and GATA-3 strong intensity. The strong intensity was more prevalent in specimens that were obtained from patients over 50 years. There were no relationships between GATA-3 expression with the nuclear grade, presentation of GCDFP15, size of the tumor, lymph node involvement, and lymphovascular invasion. In terms of GCDFP15 expression, the presentation of this marker had a relationship with age, and GCDFP15 expression in patients who are younger than 50 years old, is lower than in another group.

Triple-negative breast cancer (TNBC) has no standard and correct diagnostic profile (19). Triple-negative breast cancer is considered the most aggressive and heterogeneous malignancy involving the breast (20, 21). TNCB with a lack of expression of hormone receptors such as ER (Estrogen Receptors) and PR (Progesterone Receptors), lack of expression of HER-2 (human epidermal growth factor receptor-2), and expression of some breast tissue genes (EGFR, Cyclin-E, CK17, CK15) is determined. TBNC includes about 13-17% of all breast cancer cases, and in general, the survival rate of patients in this category of disease is lower than other groups (average survival of 5 years) and patients always have a worse prognosis than other groups (22, 23). In TNBC patients, the rate of metastasis to distant tissues such as the brain (30%), lung (40%), and liver (20%) is higher than lymph node involvement (10%) and bones (10%). It seems that the cause of bad prognosis in these patients is the involvement of vital tissues in these patients (24, 25). This issue makes this study important because finding parameters for TNBC can increase diagnosis and prevent metastasis.

In a study that was conducted by Yang* et al. *on 64 women with metastatic and primary breast cancer, it was found that GATA-3 to be more sensitive than GCDFP15 in diagnosis of breast cancer. When GATA-3 and GCDFP15 were used simultaneously, the sensitivity was improved (16). In the current study, it was observed that GATA-3 was positive in 90% of specimens and it shows that GATA-3 is a good marker for TNBC diagnosis. GCDFP15 was positive in 46% of cases. So, GATA-3 in TNBC is more prevalent than GCDFP15. In a study done by Ni *et al.*, it was found that GATA-3 to be positive more frequently than GCDFP-15 in women with invasive breast cancer. Also, they concluded a high sensitivity for GATA-3 for detection of nodal metastases and distant metastases, with good concordance with primary tumors (26). In the present study, it was observed that there were no associations between GATA-3 positivity and lymph node involvement or lymphovascular invasion. These findings were different from the findings reported by Ni* et al. *and Yang* et al.* Maybe these differences were due to the difference in the population of the studies. Those studies were performed on patients who were involved with primary or metastatic breast cancer of all types but the current study was performed on TNBC. Maybe in patients with TNBC, GATA-3 has no relationship with lymph node involvement, lymphovascular invasion, or other tumor parameters but, for other types of breast cancer, this marker is a clue for the diagnosis of lymph node involvement and distant metastasis. So, because there are few studies on this topic, future studies should be performed on the expression of GATA-3 in different types of breast cancer as a diagnostic marker for lymphovascular invasion, lymph node metastasis, or other tumor parameters.

Ni* et al. *conducted a study on patients with metastatic breast cancer and primary TNBC. They found about 90% of cases to be positive for GATA-3, which was similar to our study (26). 

With reported overall sensitivity ranging from 10 to 79 percent, while GCDFP-15 is now considered as an available and one of the best immunohistochemical markers for diagnosis of metastatic breast cancer, it shows a significant decreased sensitivity in TNBC. Therefore, it is challenging and crucial to clinically identify the site of tumor development , particularly for those metastatic tumors without a prior history of breast cancer (13, 27-29). Wang* et al. *evaluated all gene expression of TNBC in a multi-centric study and they found that GCDFP-15 is not a common marker in patients with TNBC (30). In the current study, we obtained similar finding to Wang *et al.*’s study. 

## Conclusion

GATA-3 and GCDFP-15 may be two markers for the diagnosis of triple-negative breast cancers with different prevalences. GATA-3 expression is more frequent than GCDFP-15 positivity. These markers seem to have no relationship with tumor markers such as lymphovascular invasion, lymph node metastasis, mass size, nuclear and histologic grade. Both markers may be associated with age. The frequency of positivity of these markers may be higher in patients older than 50 years. Further studies on GATA-3 and GCDFP-15 would be recommended in the future using more cases and different types of breast cancer.

## Conflict of Interest

The authors declared no conflict of interest.
